# Functional Versatility of AGY Serine Codons in Immunoglobulin Variable Region Genes

**DOI:** 10.3389/fimmu.2016.00525

**Published:** 2016-11-22

**Authors:** Thiago Detanico, Matthew Phillips, Lawrence J. Wysocki

**Affiliations:** ^1^Department of Biomedical Research, National Jewish Health, Denver, CO, USA; ^2^Department of Immunology University of Colorado School of Medicine, Denver, CO, USA

**Keywords:** B cell, V-genes, somatic hypermutation, autoantibodies, antiviral immune response, autoimmunity, lupus erythematosus, systemic

## Abstract

In systemic autoimmunity, autoantibodies directed against nuclear antigens (Ags) often arise by somatic hypermutation (SHM) that converts AGT and AGC (AGY) Ser codons into Arg codons. This can occur by three different single-base changes. Curiously, AGY Ser codons are far more abundant in complementarity-determining regions (CDRs) of IgV-region genes than expected for random codon use or from species-specific codon frequency data. CDR AGY codons are also more abundant than TCN Ser codons. We show that these trends hold even in cartilaginous fishes. Because AGC is a preferred target for SHM by activation-induced cytidine deaminase, we asked whether the AGY abundance was solely due to a selection pressure to conserve high mutability in CDRs regardless of codon context but found that this was not the case. Instead, AGY triplets were selectively enriched in the Ser codon reading frame. Motivated by reports implicating a functional role for poly/autoreactive specificities in antiviral antibodies, we also analyzed mutations at AGY in antibodies directed against a number of different viruses and found that mutations producing Arg codons in antiviral antibodies were indeed frequent. Unexpectedly, however, we also found that AGY codons mutated often to encode nearly all of the amino acids that are reported to provide the most frequent contacts with Ag. In many cases, mutations producing codons for these alternative amino acids in antiviral antibodies were more frequent than those producing Arg codons. Mutations producing each of these key amino acids required only single-base changes in AGY. AGY is the only codon group in which two-thirds of random mutations generate codons for these key residues. Finally, by directly analyzing X-ray structures of immune complexes from the RCSB protein database, we found that Ag-contact residues generated *via* SHM occurred more often at AGY than at any other codon group. Thus, preservation of AGY codons in antibody genes appears to have been driven by their exceptional functional versatility, despite potential autoreactive consequences.

## Introduction

Although DNA mutations are a cornerstone of species evolution and adaptation, somatic mutagenesis is generally suppressed in eukaryotes. An important exception is the somatic hypermutation (SHM) of antibody variable (V) region genes, which is initiated by activation-induced cytidine deaminase (AID) and provides the structural basis of affinity maturation during physiological Ab responses. However, a byproduct of SHM is the generation of B cells with autoreactive receptors. Although, autoreactive B cells are normally eliminated by immune self-tolerance mechanisms, they sometimes escape censorship to participate in systemic autoimmune diseases such as lupus erythematosus (SLE). In particular, Abs directed against nuclear antigens (ANA) are a hallmark of SLE.

In prior studies involving a spontaneous mouse model of SLE, we have shown that many ANA arise by SHM of non-autoreactive B cells and that this conversion was strongly correlated with SHM of complementarity-determining regions (CDR) AGY Ser codons to Arg codons (1, 2). Arg residues are known to contribute substantially and often decisively to the binding energy between ANA and their nuclear targets (1, 3–11). In agreement with this, D regions that are enriched with Arg residues have a profound effect in B cell development and can induce spontaneous autoantibody production in mice (12). Moreover, AGY codons are unique in their potential to mutate to an Arg codon by any one of three different single-base changes, and the AGC trinucleotide is an intrinsically preferred target of SHM (13–16).

Curiously, our analyses of the germline repertoire of IgV-region genes revealed that these seemingly dangerous AGY Ser codons are unusually abundant in sequences specifying CDRs, a phenomenon that is conserved in human and mouse repertoires (1, 2). As such, VH, Vκ, and Vλ genes appear poised to mutate in a manner that would frequently generate antinuclear activity in the specified Ab product. Moreover, AGY Ser codons are more frequent than TCN Ser codons in germline IgV-region CDRs, a bias that does not apply to αβTCRV-region genes, which favor TCN over AGY codons (3, 17, 18). Because AGY, but not TCN, is an intrinsically preferred target of SHM, it was speculated that this AGY bias evolved to enhance targeting of SHM to antibody CDRs (17).

In this study, we asked whether the AGY serine codon bias and abundance in CDRs were highly conserved from an evolutionary perspective, and if so, whether this might be explained by a selection pressure to enhance overall CDR mutability. We found these features to be conserved in the most primitive vertebrates with an acquired immune system, but not solely because they enhance CDR mutability. Notably, the AGY triplet was abundant only in the Ser reading frame. In an unexpected twist, we found that AGY codons in antiviral antibodies were frequently mutated to codons specifying most of the amino acids that were reported to be key binding-site contact residues for antigen (Ag), as determined from more than 100 crystal structures of Ag–Ab complexes (19). Because the germline codons that gave rise to somatically generated contact residues were not determined in this study, we conducted additional analyses of published Ab–Ag crystal structures to identify germline codons that mutated to codons specifying contact residues. Our independent analyses revealed that somatic mutations in AGY codons created Ag-contact residues more often than mutations in any other synonymous codon group. As such, it appears that AGY CDR codons were preserved because of their exceptional functional plasticity in the context of SHM and affinity maturation.

## Materials and Methods

### IgV- and TCRV-Region Gene Sequences

A database of nucleic acid sequences for germline-encoded CDR1 and 2 and frameworks 1, 2, and 3 of functional Ig V-regions was extracted from www.ncbi.nlm.nih.gov/projects/igblast and compiled as described (2). All available mouse and human V genes were used in the analyses. Framework regions (FRs) and CDR sequences were defined using the Kabat and/or IMGT definitions as indicated in the text or figure legends (20–22). The framework regions (FRs) 1–3 or CDR1 and 2 sequences were fused to form a continuous sequence, and codon frequencies were calculated by the function provided at http://www.kazusa.or.jp/codon/. This approach was made possible by the fact that CDR and FR definitions begin and end with intact codons. IgVH genes from cartilaginous fishes were extracted from http://www.imgt.org/. All 12 functional genomic DNA sequences available at the time of the analyses were used to determine the average observed/expected ratios of AGY and TCN Ser codons among germline-encoded CDRs. The following sequences were used in the analyses: *Ginglymostoma cirratum* (IGHV2S1*01, IGHV2S2*01, IGHV2S3*01, and IGHV2S4*01), *Heterodontus francisci* (IGHV1S1*01, IGHV1S15*01, IGHV1S3*01, IGHV1S4*01), *Leucoraja erinacea* (IGHV1S3*01, IGHV1S4*01, and IGHV1S5*01), and *Hydrolagus colliei* (IGHV1S3*01). Nucleotide sequences encoding mouse TCRV-region CDRs (IMGT definition) were also extracted from functional V genes at http://www.imgt.org/ (20). In cases where a V gene had multiple alleles, the first listed allele was analyzed.

### Sequence Analyses

Observed over expected ratios were calculated by dividing the codon observed frequency (described above) by the expected frequency obtained from the codon use table for the species at http://www.kazusa.or.jp/codon/. Reading frame frequencies for CDR AGY triplets were determined manually, with the provision that any non-Ser AGY triplet that overlapped a FR–CDR boundary was conservatively included in the corresponding non-coding CDR Ser reading frame.

### Antiviral Antibody Sequences

Sequences of antiviral Abs were obtained from http://www.ncbi.nlm.nih.gov/nuccore/. The influenza antibody sequences were originally described by Wrammert et al. (23) and Li et al. (24). The search criteria used for the other antiviral Abs were “virus AND antibody AND *Homo sapiens* AND range: 300–800 bp” using the nucleotide database at PubMed. Sequences were chosen based on their order of appearance. The GI numbers for analyzed sequences are: Rhinovirus: 475389817, 475389820, 475389822, 475389827, 475389830, 475389834, 475389838, 475389842, 475389846, 475389853, 475389856, and 475389859. Avian Influenza: 269273439, 269273440, 269273441, 269273442, 269273443, 269273444, 269273448, 269273449, 269273450, 269273451, 269273452, 226894290, 226894291, 226894292, 226894293, 226894294, 226894295, 226894299, 226894300, 226894301, 226894302, 226894303, 311361464, 311361465, 311361466, 311361467, 311361468, and 311361469. West Nile: 207046350, 207046351, 207046352, 207046353, 207046354, and 207046355. Dengue: 46009632727, 46009632730, 46009632735, and 46009632737. Hepatitis A, B, and C: 7012696, 7012699, 7012701, 7012704, 7012706, 7012709, 18042112, 18042114, 18042116, 18042118, 4837672, 4837674, 4837676, 4837678, 4837680, 4837682, 4837684, 4837686, 4837688, 4837690, 4837692, 4837694, 4837696, 4837698, 29650296, 29650298, 29650303, 29650314, 29650328, 29650334, 29650337, 29650339, 76443955, 76443957, 76443959, 76443961, 76443963, 2578112092, 2578112094, 2578112096, 2578112098, 184921, 184922, 184923, 184924, 186113, 186114, 185815, 185816, 809552, 809550, 809551, 809553, 809554, 3928209, 1657318, 1657324, 1657320, 1657326, 1657322, and 1657328. Sequences were aligned using http://www.ncbi.nlm.nih.gov/igblast/, and missense mutations were determined by alignment against the closest predicted germline IgV-region gene.

### Immune Complex Crystal Structures

Structures of Ab–Ag complexes were acquired from the database at http://www.rcsb.org/pdb/home/home.do. The search criterion used was “antibody–antigen,” and the inclusion criterion was that the Ag had to be proteinaceous. Only Ab sequences from human or mouse were analyzed. Sequences were downloaded based on their order of appearance in the RCSB *protein data bank (pdb)* database. Duplicate structures were excluded from analysis. Contact residues between Ab and Ag were calculated using *ncont* from the *CCP4* program suite with an atom to atom cutoff distance of 4 Å (25). When calculating, Ag contacts, the complete Ab heavy and light chain sequences (as written in the individual pdb files) along with the complete Ag sequence were used in the search parameter. The heavy and light chain search sequences were restricted to amino acid side-chain atoms only while contact residues in the Ag were not restricted. If more than one Ab–Ag complex was present in the asymmetric unit, only one complex was included in the analysis. A total of 46 *M. musculus* and 26 *H. sapiens* immune complexes crystal structures were analyzed and the *pdb* files for these are 4ot1, 4rrp, 4tsc, 4v1d, 4xak, 4xvu, 4zs6, 5c0s, 2dd8, 3gbn, 3lzf, 3sdy, 4fqi, 4hkx, 4m5z, 4o58, 4py8, 4r8w, 4xnm, 4yjz, 5a3i, 5dum, 4dgv, 4mwf, 4n0y, 4uta, 1eo8, 1nca, 1nma, 1qfu, 2aep, 2b2x, 2xqy, 2nr6, 2ypv, 3gi9, 3hb3, 3i50, 3mj9, 3o0r, 3rv, 3v7a, 3wfb, 3wfc, 4aei, 4cad, 4etg, 4ffv, 4gag, 4gms, 4hlz_2, 4k2u, 4lqf, 4u0r, 4m1g, 4m48, 4mhh, 4n8c, 4oii, 4okv, 4plj, 4qnp, 4qww, 4rgn, 4rgo, 4tuk, 4u6h, 4xpa, 5c0n, 5dj8, 5dlm, and 5en2.

### Statistical Analysis

Statistical analyses were performed using GraphPad Prism version 5.00 for Windows, GraphPad Software, San Diego, CA, USA, www.graphpad.com.

### Box Plot-Whiskers Graphs

Box plots with notches were created using the web tool at http://boxplot.tyerslab.com/. Center line shows the median; box limit indicates the 25th and 75th percentiles as determined by R software; whiskers extend to minimum and maximum of the values; crosses indicate sample means (26, 27). The notches are defined as ±1.58× interquartile range per square root (*n*) and represent the 95% confidence interval for each median.

## Results

### AGY Ser Codons, but Not TCN Ser Codons, Are Enriched in Germline-Encoded CDR Sequences of IgV-Region Genes

It is well established that CDR Arg residues play a major role in specifying the nuclear reactivity of ANA (3). Moreover, in spontaneous SLE, many ANA arise by SHM of non-autoreactive Abs (1, 28–31), and this is often associated with the conversion of CDR germline-encoded AGY Ser codons into Arg codons (1). At the same time, germline IgVH, Vκ, and Vλ genes have unusually high frequencies of AGY Ser codons in CDRs, and this tendency holds for both mice and humans (1–3, 17).

**Figure 1:**
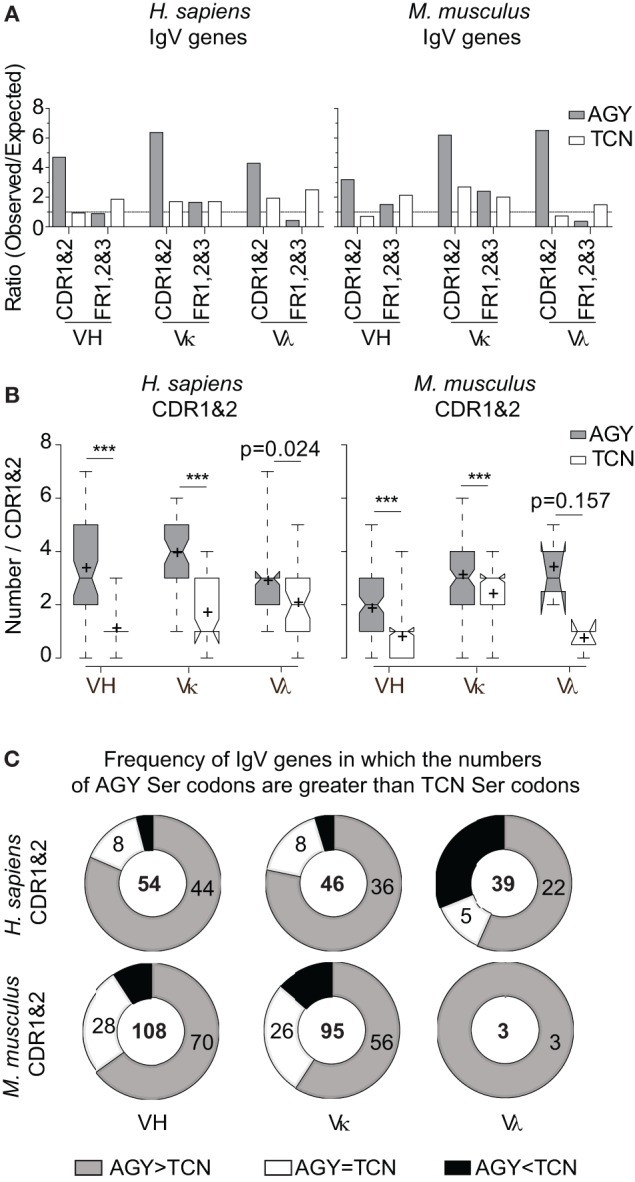
**High frequencies of AGY, but not TCN Ser codons among germline-encoded CDR sequences of IgV-region genes**. **(A)** Ratio observed/expected for AGY and TCN Ser codons in human and mouse IgV-region genes. Germline CDRs and FRs were defined using the Kabat numbering system. Expected ratio was defined by frequencies of 52,926 mouse codons and 40,662,582 human codons at http://www.kazusa.or.jp/codon/. **(B)**. Total numbers of AGY or TCN Ser codons per germline-encoded CDR sequences. Box plots were generated as indicated in Section “Materials and Methods.” Briefly, the center line indicates the median; box limits indicate the 25th and 75th percentiles; whiskers extend to minimum and maximum values, and crosses represent sample means. Notches represent the 95% confidence interval for each median. **(C)** Donut graphs represent the number of CDR1&2 AGY Ser codons minus the number of TCN Ser codons for a given gene. The gray, white, and black areas denote the number of IgV genes in which AGY Ser codon numbers are greater than, equal to, or less than TCN codon numbers respectively. Number of sequences indicated in center. *p* values were determined using a two-tailed paired *t*-test. ***p < 0.0001.

If AGY Ser codon abundance in Ab CDRs were merely due to a selection pressure to preserve Ser residues among germline-encoded V-region genes, we would expect equally high frequencies of four other serine codons (TCN). However, CDR TCN codon abundance, as defined by observed/expected ratios, was inconsistent across mouse and human VH, Vκ, and Vλ genes, reaching only 2.3-fold more than expected in the most extreme case (mouse Vκ) and less than expected in mouse and human VH genes and mouse Vλ genes (Figure 1A). Moreover, in most cases, TCN abundance was higher in FRs than in CDRs. In contrast, AGY codons were far more abundant in CDRs than expected and consistently much more so than in FRs (Figure 1A). To avoid a bias in our analyses, we took expected frequencies from codon usage tables for mouse and human genes rather than the random expected frequency of 0.016 (1/61) for a given codon. This is because the TCG codon includes the rare CpG dinucleotide, so using 0.016 would inflate the expected cumulative frequency of TCN codons, thereby reducing observed/expected ratios for TCN.

In addition to comparing observed/expected ratios for AGY and TCN codons, we also compared absolute numbers of these codons in mouse and human germline VH, Vκ, and Vλ genes. Despite a greater number of possible TCN codons, the bias favoring AGY Ser codons was still evident in all three major families of V genes for both species (Figures 1B,C). These abundance data are in agreement with data reported by Wagner et al. (17), showing that CDR AGY codons outnumber TCN codons at most CDR positions. Finally, the serine codon bias was not restricted to the idiosyncrasies of the Kabat CDR/FR definitions used in our analyses because it also applied to CDRs defined by the IMGT system (Figure S1 in Supplementary Material). Collectively, these results show that high frequencies of germline AGY serine codons in CDRs cannot be explained solely by a selection pressure favoring germline-encoded CDR serine residues.

### CDR AGY Codon Bias in Ig Genes Is the Product of an Evolutionary Selection Pressure

The frequent use of CDR AGY Ser codons among IgV-region genes from two different species (human and mouse) led us to speculate that this feature might be highly conserved in evolution. Thus, we analyzed IgVH gene sequences of cartilaginous fishes (class *Chondrichthyes*), which are descendants of the most ancient species with an adaptive immune system. The immune systems of species in this class share major features with those of mammals, including SHM, although not class switch recombination (32, 33). Our analysis of germline VH sequences from four *Chondrichthyes* species indicated that, as in mice and humans, AGY but not TCN Ser codons were enriched in germline-encoded CDR sequences (Figures S2A,B in Supplementary Material). Thus, the CDR AGY codon bias is a highly conserved feature of IgV-region genes. A similar trend was also observed in several other less distant species, by Jolly et al. (18).

### Preferential Use of AGY Triplets in the Ser Codon Reading Frame

**Figure 2:**
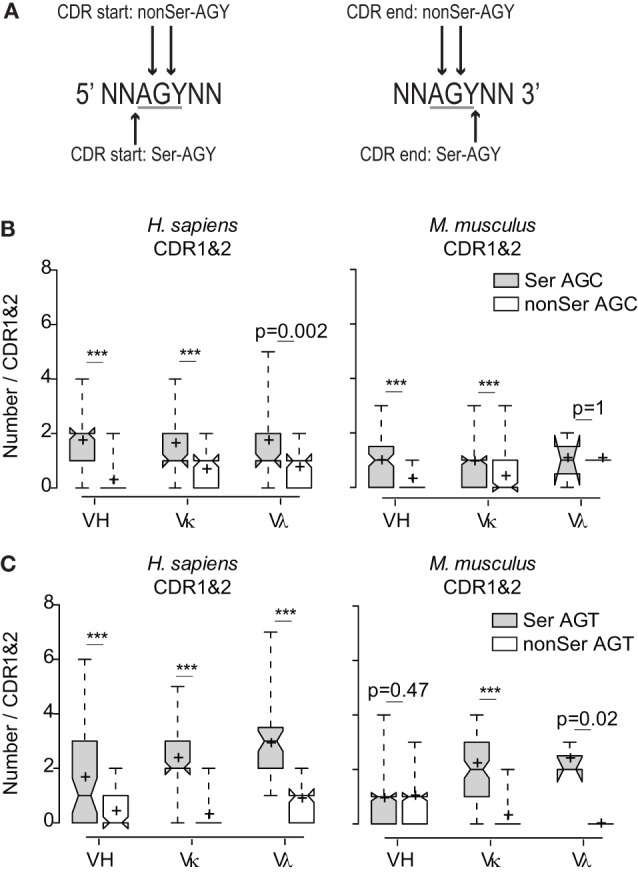
**Preferential use of the AGY triplets among CDR sequences in the Ser reading frame**. **(A)** Schematic of how AGY triplets in the different reading frames were determined at CDR boundaries. AGY triplets at CDR boundaries were counted in non-coding frames if one or two bases were located in the CDR. **(B)** Numbers of in-frame Ser AGC codons compared to combined numbers of AGC triplets in two non-coding frames. **(C)** Same analysis as in **(B)** applied to AGT. Box plots and whiskers were defined in Figure 1 and in Section “Materials and Methods.”

Because the AGC triplet has been shown to be an intrinsically preferred target for AID-dependent SHM (13, 15, 16, 34, 35), it is plausible that high frequencies of CDR AGY codons resulted solely from an evolutionary pressure to ensure high somatic mutation frequencies in CDR sequences during immune responses. This would be consistent with the fact that αβTCR genes do not share the CDR AGY abundance and bias features with Ig genes (17, 18) (Figures S2C,D in Supplementary Material). If CDR AGY codons were preserved solely to enhance mutability, we would predict that AGY triplets would be equally frequent in all three reading frames. However, this was not the case. Even when only one AGY base was required to be contained within a CDR for inclusion in the non-coding CDR frame counts, AGY triplets in the Ser reading frame were nearly always more frequent than the combined frequencies of those in the two other reading frames (Figures 2A–C). This trend also held for AGC triplets contained within the context of the extremely mutable AGCT sequence (16, 36) (Figures S3A,B in Supplementary Material). Finally, the intrinsically mutable AGC triplet was consistently more frequent in the Ser reading frame than was the combined frequency for GCT triplets in all three reading frames (AGC on opposite strand), the only exception being the small mouse Vλ gene family (Figure S3C in Supplementary Material). These results argue that the abundance of germline CDR AGY codons was not solely due to an evolutionary selection pressure for high CDR mutability *via* SHM.

### Arginine Residues in Antiviral Ab Are Often Created by SHM of AGY Ser Codons

An abundance of CDR codons that are prone to mutate to encode antinuclear Ab seemed paradoxical. However, there is speculation that a modest degree of autoreactivity may be beneficial to antiviral immune responses (37–39). For example, some viruses display host-derived nuclear material on their capsids that might enhance B cell activation or antibody efficacy due to an avidity effect (40). Therefore, we sought to determine if Arg residues are frequently generated *via* SHM in antiviral Ab. At first, we examined somatic mutations in broadly neutralizing antibodies (bNAbs) against HIV. Although we found that somatic mutations in AGY codons frequently produced Arg codons in these Abs, the results were not easily interpreted because overall mutation frequencies were extremely high, and in many cases CDR boundaries could not be defined due to insertions and deletions. Therefore, we extended our analysis to 298 published sequences of human antibodies against eight other virus species or subspecies. This analysis revealed frequent somatic mutations converting AGY Ser codons in CDRs to Arg codons.

**Figure 3:**
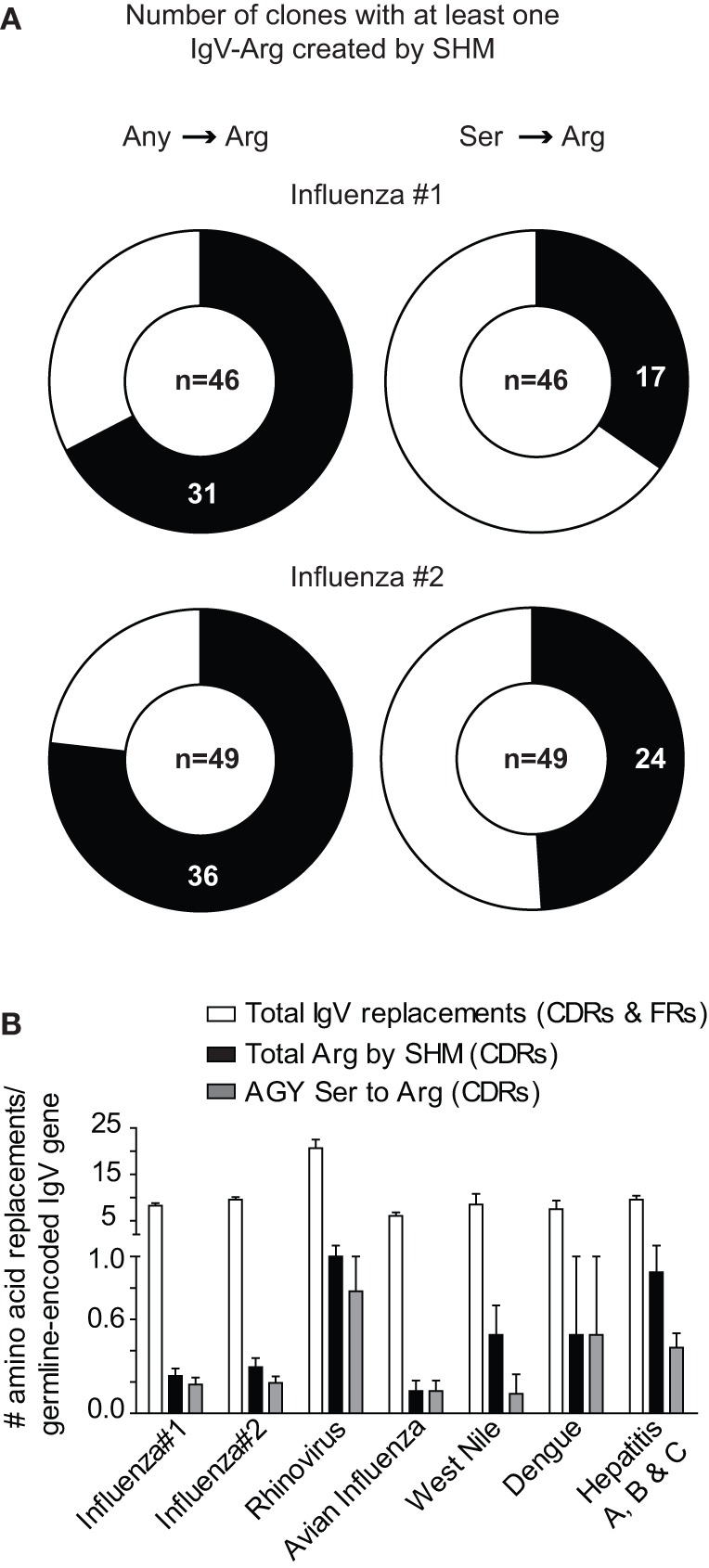
**Somatically generated Arg codons often arise at germline CDR AGY Ser codons in antiviral immune responses**. **(A)** Sequences and analyses from two studies of anti-H1N1 antibodies, as described in Section “Materials and Methods.” Heavy and light chains for a particular clone were combined to generate data for the graphs. The data combine the results of CDR and FR analyses. Any → Arg indicates a mutation at any non-Arg codon that gives rise to an Arg codon. Ser → Arg indicates an AGY Ser codon to Arg codon mutation. Numbers inside graphs indicate number of clones that were analyzed (heavy plus light chain). **(B)** Bars represent the average number of indicated replacement mutations among antiviral antibodies (heavy or light chain genes). Influenza #1 (*n* = 92), Influenza #2 (*n* = 98), Rhinovirus (*n* = 12), Avian Influenza (*n* = 27), West Nile (*n* = 6), Dengue virus (*n* = 4), Hepatitis A, B and C (*n* = 59).

In two human studies involving the H1N1 influenza virus (23, 24), 17 out of 46 and 24 out of 49 antibodies had at least one AGY Ser to Arg amino acid replacement resulting from SHM (Figure 3A). Arg replacement mutations in CDR sequences accounted for 2.9 and 3.1% of all V-region gene missense mutations (CDRs and FRs) in the two studies, with replacements at germline AGY codons comprising most of these (2 and 2.23%). A similar trend was observed in antibodies against hepatitis A, B, and C, rhino, dengue, avian influenza, and West Nile viruses. CDR Arg mutations accounted for 2.4–9.4% of all missense mutations in V-region genes for these antibodies, most of which (1.5–6.6%) occurred at germline CDR AGY codons (Figure 3B; Table 1).

**Table 1:** **Amino acid replacements *via* SHM of CDR AGY Ser codons**.

Immunogen	Asn (%)	Gly (%)	Thr (%)	Arg (%)	Others (%)	#CDR AGY SHM
Influenza^a^	22^c^	16^c^	19^c^	11	32	142
Influenza^b^	30^c^	12	23^c^	16	19	107
West Nile	20	0	60^c^	20	0	5
Dengue	14	0	43^c^	29	14	7
Rhinovirus	7	0	15	26	52	27
Avian Influenza	50^c^	0	17	33	0	12
Hep. A, B, and C	22^c^	18	18	20	22	72

*^a^Antibody sequences from Wrammert et al. (23)*.

*^b^Antibody sequences from Li et al. (24)*.

*^c^Amino acid replacements that occurred more often than Arg replacements at CDR AGY Ser codons*.

### CDR AGY Codons Frequently Mutate to Produce Codons for Key Ag-Contact Residues in the Ab-Binding Site

Our analyses of somatic mutations in antiviral Ab led to an unexpected finding: CDR AGY Ser codons frequently mutated to Asn, Thr, and Gly codons in addition to Arg codons. Most of these mutations occurred by single-base changes, predominantly at the central base in the AGY triplet (Table 2), which is the position that is preferentially targeted by AID (13). In many cases, mutations to these alternative codons, particularly those for Asn and Thr, were more frequent than to Arg codons. For example, in anti-influenza Abs, CDR AGY mutations to Asn and Thr codons were each approximately twice as frequent as mutations to Arg codons. These observations were particularly revealing because in their analyses of numerous crystal structures of Ab–Ag complexes, Raghunathan et al. (19) identified Asn, Thr, Arg, Gly, Ser, Asp, and Tyr as key (i.e., most frequent) Ag-contact residues.

**Table 2:** **Base distribution of somatic mutations in CDR AGY Ser codons**.

Immunogen	AGY (%)	AGY (%)	AGY (%)	2 changes (%)	3 changes (%)
Influenza^a^	12	53	11	20	4
Influenza^b^	11	52	15	20	2
West Nile	0	80	20	0	0
Dengue	15	57	14	14	0
Rhinovirus	0	22	19	52	7
Avian Influenza	0	67	33	0	0
Hep. A, B, and C	12	35	18	35	0

*^a^Antibody sequences from Wrammert et al. (23)*.

*^b^Antibody sequences from Li et al. (24)*.

**Figure 4:**
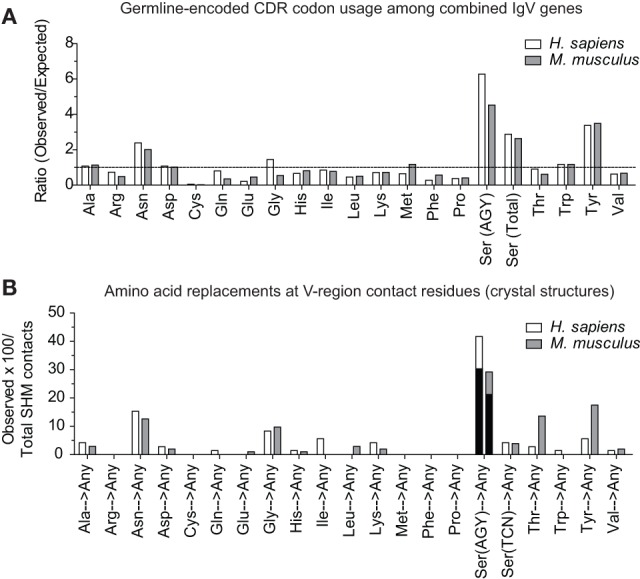
**CDR AGY Ser codons play a key role in affinity maturation**. **(A)** Ratio observed over expected for synonymous codons in CDR sequences of combined IgV genes (VH, Vκ, and Vλ). **(B)** Percentage of the total contact residues that were created by SHM in V-region sequences only. Each data set represents a germline-encoded codon given rise to any contact residue. Black bars represent the percentage of AGY Ser codons that gave rise to a key contact residue defined by Raghunathan et al. (19).

In the report by Raghunathan and colleagues, it was not clear which contact residues were generated by SHM. To determine if residues frequently generated by SHM of AGY Ser codons are associated with Ab affinity maturation, we analyzed 72 (46 mouse and 26 human) Ab–Ag crystal structures available in the RCSB *protein data bank (pdb)* database, identified predicted Ag-contact residues, and searched IgBLAST to distinguish those that were germline-encoded from those that were somatically generated. When mouse and human data where combined, the seven most frequent Ag-contact residues were Arg, Asp, Asn, Gly, Ser, Thr and Tyr (Figure S4 in Supplementary Material). This result is identical to that of Raghunathan et al. (19), even though only 4 of the 72 structures we analyzed were also analyzed by them. Yet, we found that only three (Asn, Ser, and Tyr) of those seven residues (Arg, Asn, Asp, Gly, Ser, Thr, and Tyr) were present at higher frequencies than expected within CDRs of mouse and human germline IgV-region genes (Figure 4A). Importantly, amino acids resulting from SHM accounted for only 10–23% (average 14.7%) of all Ag-contact residues (Table 3 footnotes; Figure S4 in Supplementary Material). This is relevant to our conclusion regarding AGY versatility because it means that the seven key Ag-contact residues were largely defined by germline-encoded contacts; yet four (Asn, Arg, Gly, and Thr) of the seven most abundant contact residues arise frequently from somatic mutations at CDR AGY codons.

**Table 3:** **Amino acid replacements due to somatic mutation of germline AGY Ser codons**.^a^

	Contact mutations at AGY^b^		% of all contact mutations^c^	
	Human	Mouse	Human (%)	Mouse (%)
Arg	4	7	5.55	6.73
Asn	5	9	6.94	8.65
Gly	0	1	0	0.96
Thr	6	5	8.33	4.81
Others	15	8	20.83	7.69

*^a^Data from 26 human and 46 mouse crystal structures of Ag–Ab complexes*.

*^b^V-region contact residues arising from SHM of AGY Ser codons. Numbers expressed in absolute numbers. Total contact residues analyzed were 317 (human) and 886 (mouse). Total contact residues that were associated with SHM of a V-region codon were 72 (human) and 104 (mouse)*.

*^c^Percentage of total somatically generated contacts residues that arose from mutation of AGY Ser codons*.

**Figure 5:**
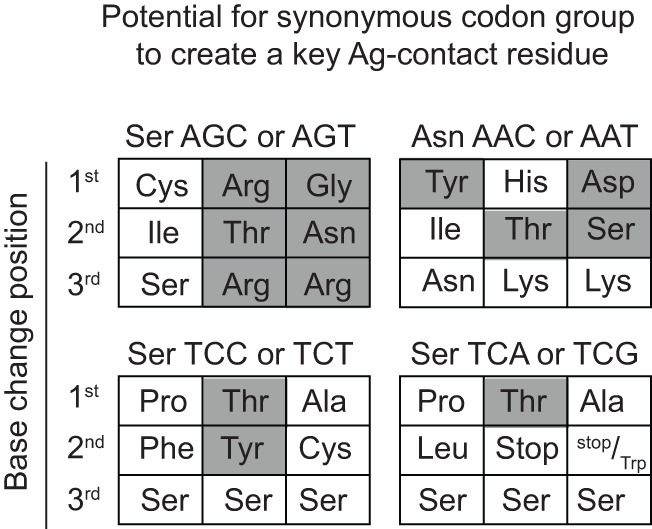
**AGY Ser codons plasticity**. Probability of creating a key non-synonymous contact residue by one nucleotide change. Filled gray boxes indicate a key Ag-contact residue as defined by Raghunathan et al. (19). White boxes indicate a synonymous change, a non-key contact residue (defined in the text) or a stop codon.

For somatically generated contact residues, mutations at AGY Ser codons were the most abundant by far, and occurred ~2–3 times more often than mutations at AAY Asn codons (Figure 4B), the second most consistently mutated codon group. Most importantly, AGY Ser codons mutated to contact residues more often than any other codon group (Figure 4B), and a large proportion of these (~70%) were those defined as key Ag-contact residues. AGY mutations to codons for Arg, Asn, and Thr were the most consistent, and this was true for both contact and non-contact residues (Table 3 and data not shown). AAY triplets are also intrinsically preferred targets of SHM (13, 15, 16). However, when considering the potential to mutate to 1 of the 6 non-synonymous key contact residues (Arg, Asn, Asp, Gly, Ser, Thr, and Tyr), AGY Ser codons are able to do so *via* 12 out of 18 possible single-base changes. For AAY (Asn), this occurs with 8 out of 18 base changes, and for TCN, it occurs with only 6 out of 36 base substitutions (Figure 5), a result that is in agreement with the observation by Chang and Casali that CDR, but not FR sequences, are prone to acquire replacement mutations upon random point mutation (41). Collectively, the results of these analyses indicate that AGY codons contribute to Ab affinity both directly, by encoding a Ser residue, and indirectly due to the ease with which they mutate to encode other residues beneficial to the process of Ab affinity maturation. We believe this is the most straightforward explanation for the conservation of AGY codon abundance in CDRs of germline IgV-region genes.

## Discussion

Our study is rooted in the observation that germline-encoded IgV-region genes have inordinately high frequencies of AGY Ser codons, particularly in CDRs. This applies across mouse and human VH, Vκ, and Vλ germline genes but not to αβTCR genes. We show that this trend is conserved even in cartilaginous fishes.

AGY Ser codons are potentially dangerous because they easily mutate to generate Arg codons with an associated potential to impart antinuclear activity to the respective antibody (1, 3). This raises a paradox because TCN Ser codons do not have this propensity and yet are far less abundant in Ab V region genes and specifically in CDRs. Wagner and colleagues originally hypothesized that this bias toward AGY Ser codons in CDRs was due to a selection pressure to constrain AID motifs to Ag-binding regions of the B cell receptor (17). While this is plausible, our data reveal that this explanation alone cannot account for CDR AGY codon abundance because CDR AGY triplets occur predominantly in the Ser reading frame, even though AID is blind to the translational reading frame. Because TCA and TCG can mutate to stop codons by single-base changes, it is plausible that high CDR AGY/TCN ratios are due in part to selection against these codons. This may hold for TCG which had a low observed/expected ratio, but apparently not for TCA, which had an observed/expected ratio of greater than one, even though it can mutate to a stop codon by two different single-base changes. Overall the observed/expected ratios for TCN codons were greater than one in CDRs. Finally, if there was selection pressure against TCN due to the stop codon potential, we would expect that TCN would be underrepresented in CDRs relative to FRs because there is a bias for increased mutation in CDRs that cannot be explained solely by triplet sequences (13). However no such bias was seen for the Vκ genes of either species (Figure 1A).

In view of reports that a measure of autoreactivity may be beneficial in the context of some antiviral antibody responses, we asked whether somatic mutations that generate Arg codons arise frequently in antiviral antibodies, and specifically at AGY codons (37–39, 42–48). While it was not possible to clearly address this question in the context of broadly neutralizing anti-HIV antibodies, we were able to address it in the context of Abs directed against six different viruses. In every case, mutations producing Arg codons were present, often in abundance, and predominantly at AGY codons.

This result alone, however, did not provide insight regarding the potential value of antinuclear activity generated *via* SHM. Our analyses of X-ray structures of Ag–Ab complexes also did not shed light on this question because we examined complexes involving only protein Ags. However, our sequence analyses of antiviral antibodies did reveal a considerable variation in the relative frequency with which an AGY codon mutated to encode an Arg codon versus a codon for Asn or Thr. Based on triplet mutability indices and base preference targeting by AID, we would expect a ~2:1 ratio favoring mutations to Asn/Thr codons over mutations to Arg codons (13). Overall, the Asn + Thr/Arg ratio was 2.7:1 among combined antiviral antibodies, suggesting some selection pressure against Arg. However, there was considerable variation among different antiviral antibodies. For example, while the 2:1 ratio closely approximated that seen for antibodies to hepatitis virus, the ratio was ~3.5:1 for antibodies against influenza. It is unclear whether deviations from the expected ratio are due to the autoreactive properties of CDR Arg residues or simply due to direct Ag-contact considerations. Arg is larger than Asn or Thr, such that replacing Ser with Arg may impede Ag engagement more often due to steric effects. Results of our analysis together with those of a prior study by Raghunathan et al. (19), however, indicate that Arg residues in Ab V regions frequently make contact with protein Ags. Thus, regardless of whether Ab affinity for nuclear Ags is beneficial to some viral immune responses, somatic mutations that produce Arg codons at germline CDR AGY codons can be beneficial to the development of high-avidity antibodies.

We also found, unexpectedly, that AGY codons in antiviral Abs mutated frequently to codons for most of the other amino acids that were identified as key Ag-contact residues in the Ab-binding site (19). Only a single-base change was required to generate codons for most of these key residues. Among the antiviral Abs we analyzed, point mutations in AGY that generated codons for these key residues occurred predominantly at G and C, which are the major initiation sites for SHM by AID.

Finally, upon analyzing X-ray structures of immune complexes involving protein Ags, we found that Ag-contact residues created by SHM occurred more frequently in AGY codons than in any other synonymous codon group. And this was also true for the key contact residues defined by Raghunathan and colleagues primarily on the basis of germline-encoded contacts. Notably, all of these key contact residues are polar or charged. Polar and charged amino acids are preferentially found on solvent-exposed surfaces of all proteins. Additionally, small polar amino acids are often favored in loop regions where they contribute both to flexibility and direct contacts with other protein ligands due to small side chains with minimal steric barriers. Polar residues, such as Ser, Asn, and Thr, can act as both hydrogen bond donors and acceptors thus making them ideal residues to accommodate a number of different binding landscapes: they can form hydrogen bonds with other polar residues as well as basic and acidic residues (49, 50). Serine, being one of the smallest amino acids, is perhaps the most compliant residue. Other small amino acids, such as Cys and Ala, would be less favored do to unwanted disulfide bond formation (Cys) or lack of hydrogen bonding (Ala).

Mutation of Ser to another small to midsize polar residue, such as Thr, Gly, and Asn, maintains most of the binding plasticity of serine while potentially adding additional binding energies from either increased van der Waals interactions, stronger hydrogen bond strength due to decreased hydrogen bond length, or both. Thus serine is an ideal residue for contributing to binding on its own, while, at the same time, being an ideal starting point for mutation to other small polar groups. Replacing Ser with a larger amino acid such as Arg during SHM, while beneficial in some cases, may come with a higher probability of disrupting the interaction between Ab and Ag. This may account for the high ratio of Asn and Thr to Arg replacement mutations at CDR AGY codons of influenza antibodies. It is notable that unlike the case for AGY codons, random base substitutions in TCN Ser codons often lead to large hydrophobic residues or to less favorable residues, such as Ala (non-polar) and Cys (potentially disruptive). In sum, the fact that Ser is one of the seven major Ag-contact residues, together with the ease with which AGY Ser codons can mutate to four more of these residues, provides the most straightforward explanation of why AGY codon abundance in Ab CDRs is conserved from sharks to humans.

## Author Contributions

TD, MP, and LW: data acquisition, analysis, interpretation, and manuscript preparation.

## Conflict of Interest Statement

The authors declare that the research was conducted in the absence of any commercial or financial relationships that could be construed as a potential conflict of interest.
