# Supplementary material for: Functional Versatility of AGY Serine Codons in Immunoglobulin Variable Region Genes
Source: Front Immunol. 2016 Nov 22;7:525. doi: 10.3389/fimmu.2016.00525 (PMC5118421; doi:10.3389/fimmu.2016.00525)
Supplement: Supplementary file 1 [file Image_1.PDF]

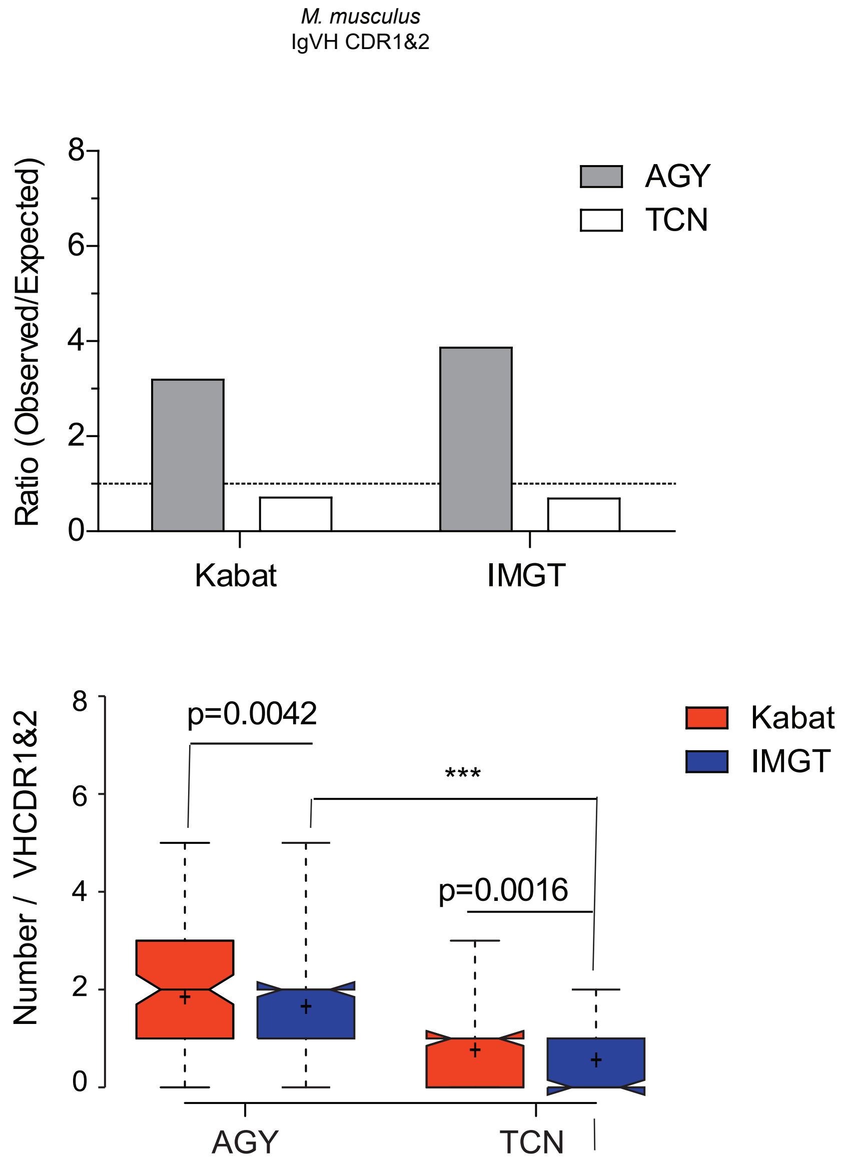

Supplemental Figure 1: AGY codon bias applies to CDRs defined by Kabat and IMGT systems. Top graph, observed/expected ratio as described in Figure 1. Bottom graph, total numbers of AGY and TCN Ser codons. Center lines show the medians; box limits indicate the 25th and 75th percentiles as determined by R software; whiskers extend to minimum and maximum values; crosses represent sample means. n = 108. Mouse VH genes were conservatively chosen because they showed the weakest trend in our analyses (Fig. 1). P values were determined using paired t-test. *** indicates p<0.0001
