# Supplementary material for: Functional Versatility of AGY Serine Codons in Immunoglobulin Variable Region Genes
Source: Front Immunol. 2016 Nov 22;7:525. doi: 10.3389/fimmu.2016.00525 (PMC5118421; doi:10.3389/fimmu.2016.00525)
Supplement: Supplementary file 2 [file Image_2.PDF]

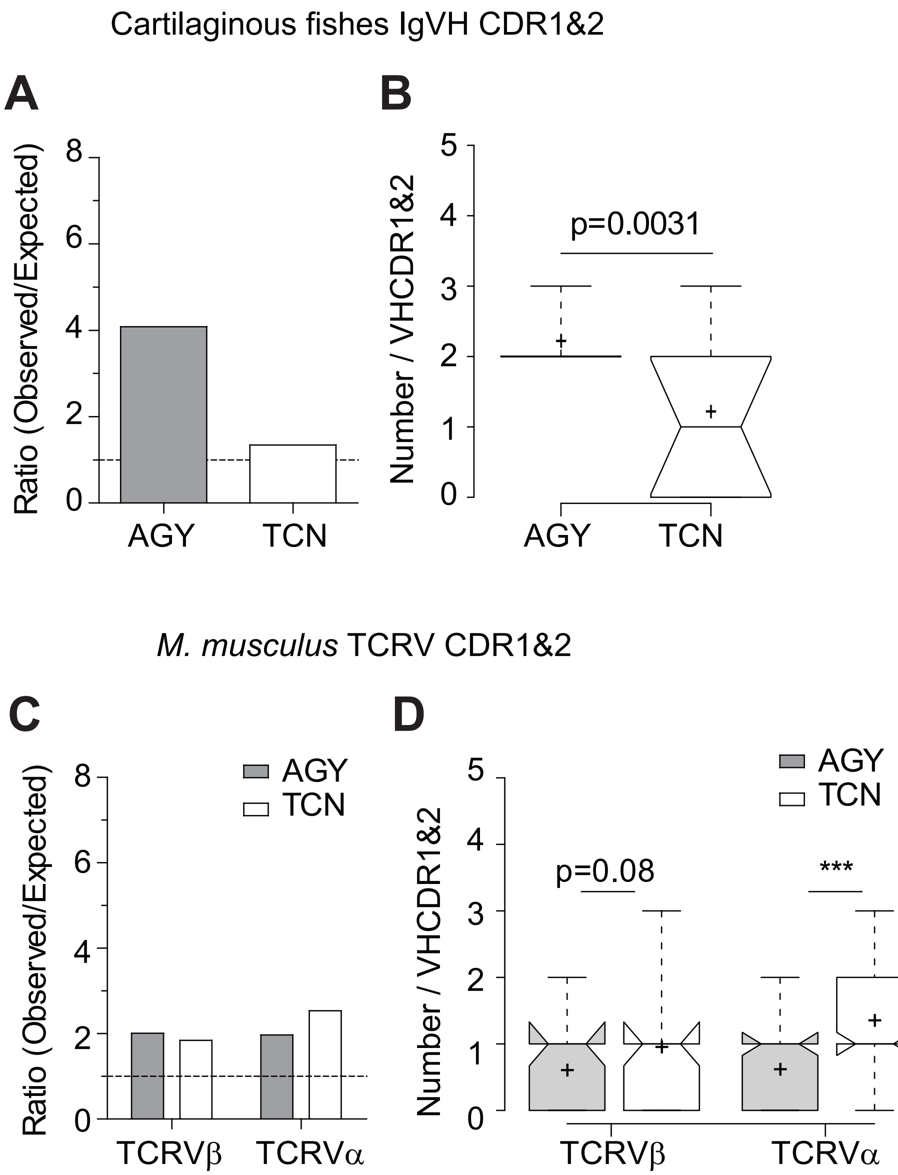

Supplemetal Figure 2: Evolutionarily conserved and restricted use of CDR AGY Ser codons in IgV genes. (A) IgVH genes from Cartilaginous fishes were analyzed. Observed/expected ratios for AGY and TCN Ser codons were calculated as in Figure 1 legend except that CDR boundaries were defined by the IMGT system. (B) Absolute numbers of AGY and TCN Ser codons per VHCDR1 and 2, n=12. (C) Mouse αβTCRV CDR sequences were obtained as described in Materials and Methods and analyzed as described for IgV-region genes. Observed/expected ratios for AGY and TCN Ser codons were calculated as above, using the IMGT system. (D) Absolute numbersof AGY and TCN Ser codons observed in CDR1 and CDR2 sequences. The number of analyzed sequences were: M. musculus TCRVβ (n=23), TCRVα (n=83). P value were determined with a two-tailed paired t-test. Box plots and whiskers extensions are defined in Figure 1.
