# Supplementary material for: Functional Versatility of AGY Serine Codons in Immunoglobulin Variable Region Genes
Source: Front Immunol. 2016 Nov 22;7:525. doi: 10.3389/fimmu.2016.00525 (PMC5118421; doi:10.3389/fimmu.2016.00525)
Supplement: Supplementary file 3 [file Image_3.PDF]

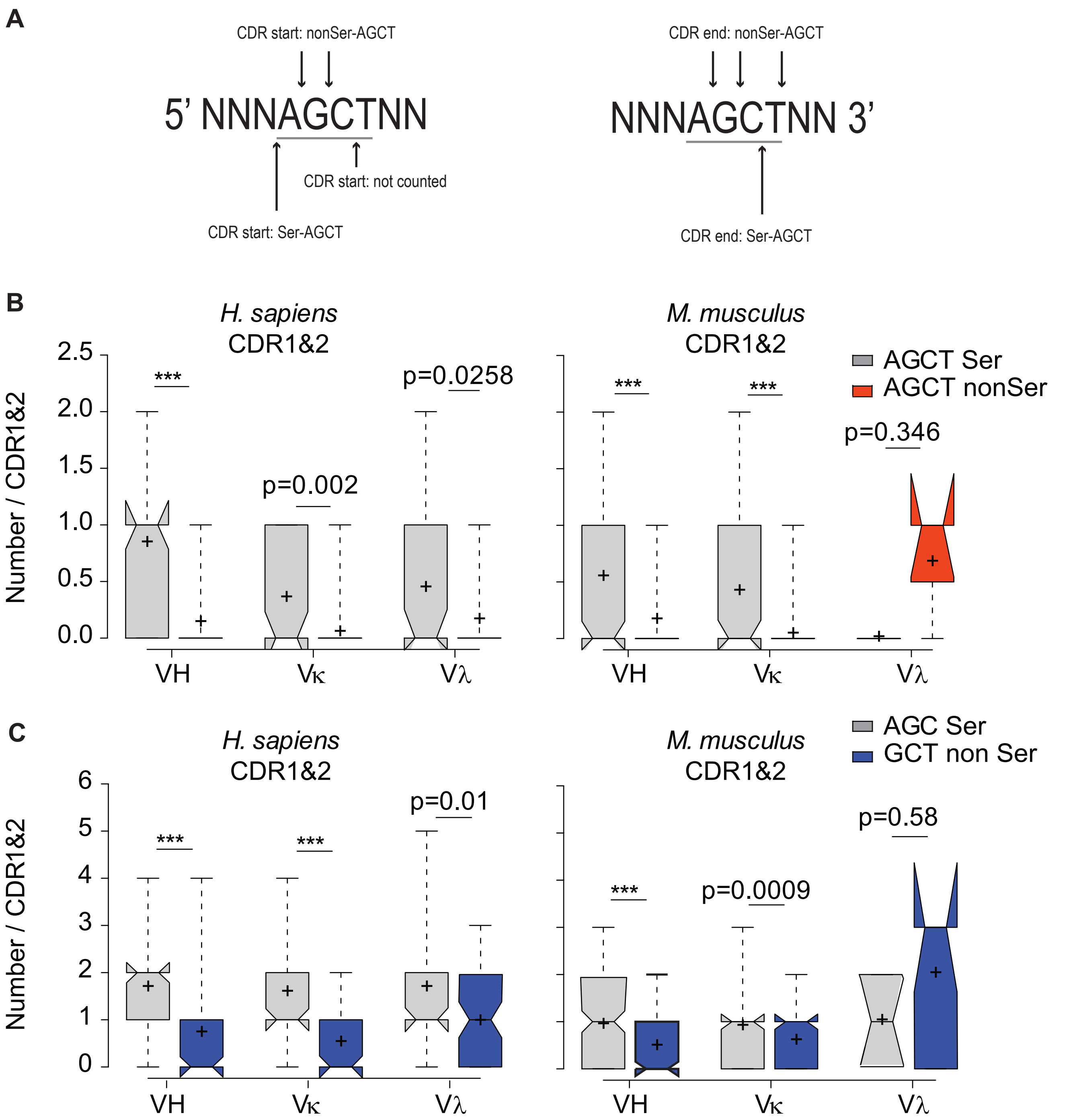

Supplemental Figure 3: Preferential use of the AGY triplets among CDR sequences in the Ser reading frame. (A) Schematic of the AGCT analysis. (B) Numbers of AGCT sequences in the Ser reading frame versus combined numbers in the two noncoding reading frames. AGCT sequences at CDR boundaries were counted in the analysis if two or more bases were located within the CDR. (C) Numbers of in-frame AGC Ser codons compared to combined numbers of GCT triplets in all three reading frames except for GCT triplets preceded by an adenosine base in the Ser reading-frame (i.e. AGCT).
ACT (AGT noncoding strand) was not included in this analysis because it is not an intrinsically preferred target of SHM. P values were determined using a two-tailed paired t-test.
*** indicates $p < 0.0001$. CDRs definitions were defined using the Kabat system.
Box plots and whiskers extensions are defined in Figure 1.
