# Supplementary material for: Functional Versatility of AGY Serine Codons in Immunoglobulin Variable Region Genes
Source: Front Immunol. 2016 Nov 22;7:525. doi: 10.3389/fimmu.2016.00525 (PMC5118421; doi:10.3389/fimmu.2016.00525)
Supplement: Supplementary file 4 [file Image_4.PDF]

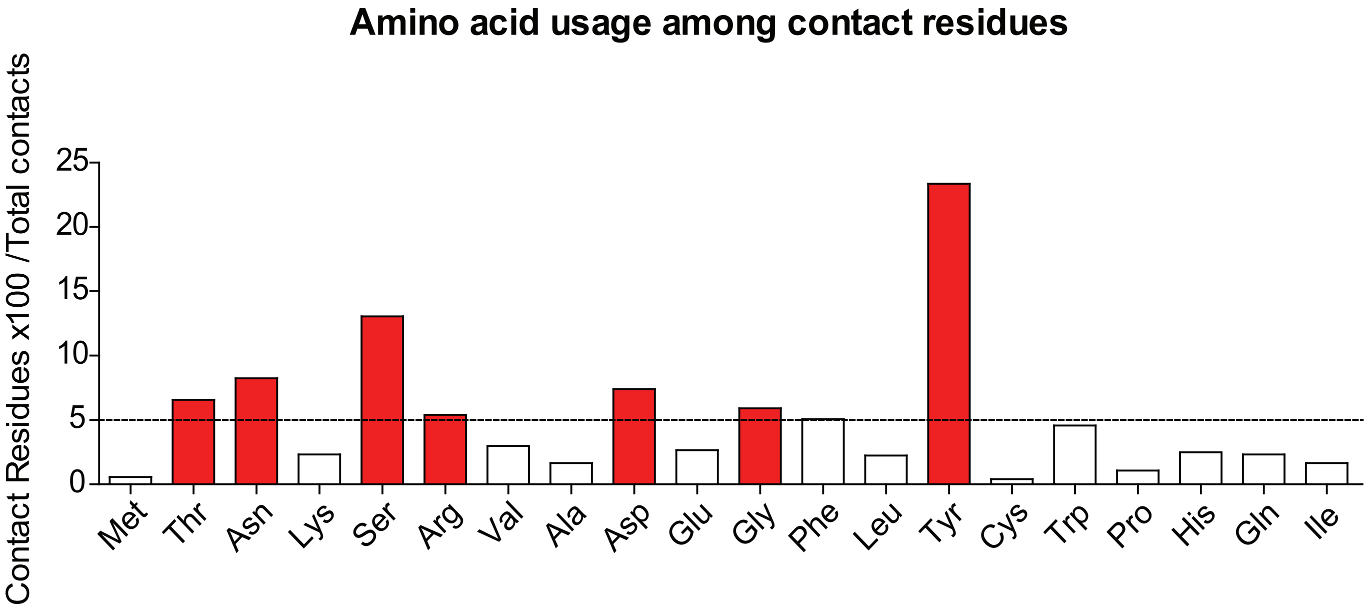

Supplemental Figure 4: Most frequent contact residues in antibody-antigen complexes. Contacts were identified in 72 crystal structures, as indicated in the Materials and Methods. Only 176 of 1203 contact residues were products of SHM. Red indicates major contact residues reported by Raghunathan et al. (20). Only 4 crystal structures were analyzed in common.
